# JAK-STAT signaling: molecular mechanism and targeted treatment in dento-maxillofacial abnormalities

**DOI:** 10.1038/s41368-025-00399-z

**Published:** 2026-03-05

**Authors:** Zihan Huang, Yiwen Cui, Wenyi Zhang, Jiachen Shen, Qinggang Dai, Siyuan Sun, Lingyong Jiang

**Affiliations:** 1 Center of Craniofacial Orthodontics, Department of Oral and Cranio-maxillofacial Surgery, Ninth People’s Hospital, Shanghai Jiaotong University School of Medicine, Shanghai Key Laboratory of Stomatology & Shanghai Research Institute of Stomatology, National Clinical Research Center of Stomatology, Shanghai, China; 2 The 2nd Dental Center, Ninth People’s Hospital, Shanghai Jiaotong University School of Medicine, Shanghai Key Laboratory of Stomatology & Shanghai Research Institute of Stomatology, National Clinical Center of Stomatology, Shanghai, China

**Keywords:** Bone development, Bone remodelling, Oral diseases

## Abstract

Dento-maxillofacial abnormalities are highly prevalent and arise as a result of a variety of etiological factors, presenting substantial challenges to treatment. The JAK-STAT signaling plays a pivotal role in dentofacial development, regulating endochondral ossification, intramembranous ossification, dental follicle formation, and enamel development. Mutations in the JAK-STAT signaling lead to syndromes associated with severe dento-maxillofacial abnormalities, including Growth Hormone Insensitivity Syndrome and Autosomal Dominant Hyper-IgE Syndrome. Corresponding mouse disease models have been developed to simulate the phenotypes observed in clinical patients and investigate their underlying mechanism. Meanwhile, several medications targeting JAK-STAT signaling, including baricitinib and imatinib, have been developed for clinical application, demonstrating significant effects in skeletal disorders such as osteoporosis and osteoarthritis, indicating promising effects in development and abnormalities of dento-maxillofacial. In this review, we aim to summarize the role of JAK-STAT signaling in the development and abnormalities of dento-maxillofacial bone, and the relevant molecules that may be utilized for clinical treatment, to shed new light on the precise treatment of dento-maxillofacial abnormalities.

## Introduction

Dento-maxillofacial abnormalities are recognized as the third oral disease globally, with prevalence rates ranging from 28.4% to 83.9% across different regions and Asia exhibiting the highest prevalence at 61.81%.^1^ All individuals affected by abnormalities present with malocclusion characteristics. Notably, 30% of patients are diagnosed with severe skeletal malformations with a handicap rate as high as 24.8%.^2^

Dento-maxillofacial abnormalities affect both the craniomaxillofacial bones and the dental-periodontal complex.^3^ In detail, abnormalities in craniomaxillofacial bones is primarily characterized by facial malformation resulting from the developmental disharmony between the maxilla and mandible, while abnormalities in the dental-periodontal complex include dental developmental anomalies, malalignment, and alveolar bone defects, resulting from developmental, inflammatory or endocrine influences.^3^ However, in most cases, early postnatal intervention is still lacking, and the treatment of dento-maxillofacial abnormalities relies on orthodontics and surgical interventions, which are costly and entail a lengthy treatment duration of at least 3 to 5 years.^4,5^ Consequently, there is a need to develop more precise and effective therapies and elucidating the pathogenic mechanisms of dento-maxillofacial abnormalities will be important in facilitating this endeavor.

The dento-maxillofacial development is a complex biological progress including intramembranous ossification, endochondral ossification, and dental follicle formation.^6^ The principal cellular participants in these processes include mesenchymal stem cells(MSCs), osteoblast lineage cells, and osteoclast lineage cells.^7^ These key cell are regulated by multiple signaling pathways such as Sonic hedgehog (Shh),^8^ Wingless/Integrated (Wnt),^9^ and Paired box (Pax).^10^ Among them, Janus kinase (JAK)-signal transducers and activators of transcription (STAT) signaling plays a critical role.^8,10,11^ In JAK-STAT signaling, JAK is phosphorylated by extracellular signals, and then goes on to phosphorylate tyrosine residues in the intracellular domain of the receptor and phosphorylated STAT (p-STAT, activated state). P-STAT dimerizes and translocates to the nucleus, where it directly regulates the gene transcription process.^12,13^

Dento-maxillofacial bone development combines both intramembranous and endochondral ossification. The JAK-STAT is important in bone formation and resorption, governing the osteoblasts, osteoclasts, and their intercellular communication. STAT1, STAT3, and STAT5 play pivotal roles in transducing signals from growth factors and cytokines, such as fibroblast growth factor (FGF)18, interleukin (IL)-6, and IL-3. Activated by extracellular signals, these STAT proteins translocate into the nucleus and regulate the expression of genes including *Runx2*, *Alp*, and *Rankl*, thereby exerting significant influence.^14–16^ Tooth development involves the transition of epithelial cells into mesenchyme, leading to gradual formation of the dental follicle. JAK-STAT signaling exerts regulatory effects on epithelial proliferation, enamel secretion, dentin formation, odontoblast differentiation, and root development, targeting key cells such as epithelial cells, ameloblasts, and odontoblasts.^17–19^

Mutations affecting JAK-STAT signaling always result in severe dento-maxillofacial abnormalities. The dominant negative mutation of STAT3, autosomal dominant hyper-IgE syndrome (AD-HIES), results in premature closure of cranial sutures.^20–22^ Patients with STAT3 gain-of-function (GOF) syndrome develop a prominent forehead, which influences intracranial pressure and cerebral development.^23^ Both autosomal dominant (AD) and autosomal recessive (AR) mutations in STAT5B cause Growth Hormone Insensitivity Syndrome with Immune Dysregulation, which is associated with dentofacial abnormalities.^24,25^ Specifically, STAT5B AD mutations lead to prominent forehead and depressed nasal bridge and STAT5B AR mutations result in microcephaly. STAT6 AD mutations, similar to STAT3, induce Hyper-IgE Syndrome, characterized by mild high palate arch, joint hyperextensibility, enamel hypoplasia and other dysmorphic features.^26,27^ Patients with STAT1 autosomal dominant gain-of-function mutations leading to Immunodeficiency 31 C exhibit developmental delay. The mutation of JAK family along with STAT2 and STAT4, can lead to systemic disorders such as Erythrocytosis and autosomal recessive Hyper-IgE syndrome, potentially affecting osteogenesis and osteoclastogenesis through alterations in the bone microenvironment.^28,29^

In this review, we summarize the role of JAK-STAT signaling in dento-maxillofacial abnormalities, and the potential therapeutic approaches targeting this signaling to elucidate the molecular mechanisms underlying dento-maxillofacial abnormalities, explore new treatment strategies, and provide reference for preventive measures against their occurrence.

## JAK-STAT signaling and dento-maxillofacial development

The formation of the dento-maxillofacial complex involves intramembranous ossification, endochondral ossification, or a combination of both. While the JAK-STAT signaling exhibits functional similarities to its role in long bone, there are distinct regulatory mechanisms in craniofacial morphogenesis. Furthermore, the dental-periodontal complex comprises specialized tissues including teeth, periodontal ligament, which are formed by unique cell populations such as ameloblasts and odontoblasts. These features are absent in classical long bone studies. Current literature lacks comprehensive reviews summarizing the specific functions of JAK-STAT signaling in this distinctive biological system.

### JAK, STAT and its negative regulator

The JAK-STAT signaling consists of three core components: cell membrane-specific receptors, JAK kinases, and STAT signal transducer molecules. The cell membrane receptors include IL-6R,^30^ IFN-γR1/2,^31^ CNTFR,^32^ and Leptin receptors, among others. Correspondingly, this signaling can be activated by various cytokines such as IL-6 family members, IFN-γ, CNTF, and Leptin.^13^ The JAK family comprises JAK1, JAK2, JAK3, and TYK2, all containing four functional domains: an N-terminal FERM domain, SH2 domain, pseudokinase domain, and canonical protein tyrosine kinase domain.^33^ The FERM and SH2 domains primarily mediate JAK-receptor binding, while the pseudokinase domain regulates the activity of the kinase domain.^12^ Extracellular signals induce receptor dimerization, bringing JAK N-termini into proximity and triggering tyrosine residue phosphorylation. Activated JAKs subsequently phosphorylate tyrosine residues on the receptor intracellular domains, creating docking sites for STAT proteins.^34^

The STAT family serves as the key signal transducer in this signaling, including STAT1, STAT2, STAT3, STAT4, STAT5A, STAT5B, and STAT6.^35^ STAT proteins contain multiple domains: an SH2 domain, N-terminal conserved domain, helical domain, DNA-binding domain, and C-terminal transcriptional activation domain.^35^ The SH2 domain is critical for phosphorylation-dependent activation, the N-terminal domain regulates STAT dimerization, the helical domain mediates nuclear import of the complex, while the DNA-binding and C-terminal domains are essential for STAT transcriptional function.^36–38^

Typically, inhibitors of JAK-STAT signaling exert their effects by targeting the SH2 domain, helical domain, or DNA-binding domain to block STAT phosphorylation, nuclear translocation, or transcriptional activity.^39^ Endogenous JAK-STAT signaling inhibitors primarily include the suppressors of cytokine signaling (SOCS) family and protein inhibitors of activated STAT (PIAS). The SOCS family has been more extensively studied. SOCS family include CIS and SOCS1-7, with CIS and SOCS1-3 specifically inhibiting JAK-STAT signaling.^40,41^ SOCS proteins function by either targeting membrane receptors or directly binding JAK kinases. Notably, JAK-STAT signaling activation induces SOCS expression, creating a negative feedback loop whereby elevated SOCS levels suppress further activation.The PIAS family includes PIAS1, PIASxα, PIASxβ, PIAS3, and PIAS4, which directly inhibit STAT function by blocking STAT-DNA binding.^42^ Emerging evidence also suggests that PIAS-mediated protein SUMOylation contributes to suppression through additional mechanisms.^43^

**Fig. 1:**
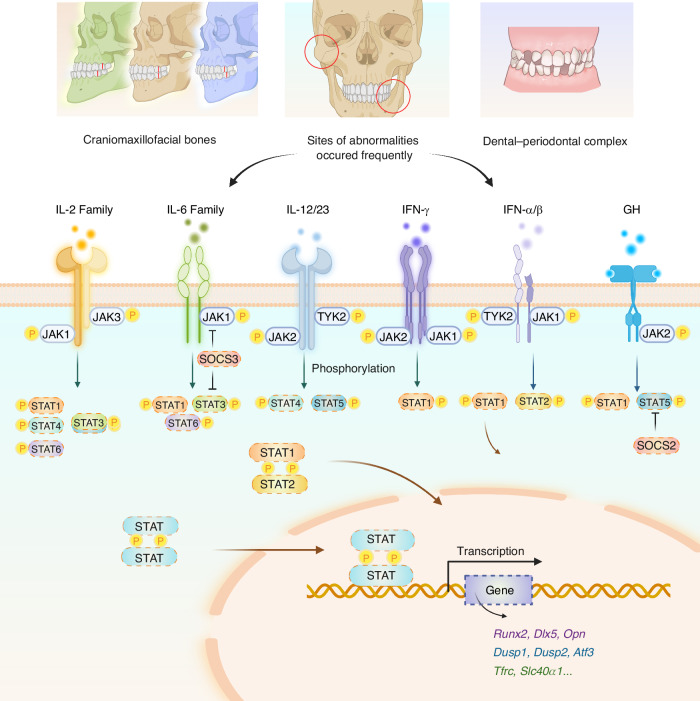
JAK-STAT signaling in dento-maxillofacial development and abnormalities. Upon binding to cell membrane surface receptors, cytokines induce receptor dimerization. Janus kinase (JAK) associates with the N-terminals of the receptors which are then brought into close proximity, leading to the phosphorylation of their tyrosine residues. The activated JAK further phosphorylates tyrosine residues on the intracellular domain of the receptor, providing docking sites for signal transducer and activator of transcription (STAT) and facilitating its phosphorylation and activation by JAK. The phosphorylated STAT (p-STAT) forms homodimers or heterodimers, which then translocate to the nucleus, where they directly bind to DNA or DNA-binding proteins to regulate the transcription of target genes. In the craniofacial field, suppressors of cytokine signaling (SOCS) primarily exert a negative regulatory effect on the JAK/STAT signaling, with related genes involved in the regulation of osteoblast and osteoclast differentiation and function (Created with bioRender.com)

In summary, the JAK-STAT signaling transduces extracellular signals and STATs mediate the gene transcription. Phosphorylation, dimerization, and nuclear translocation of STAT proteins in this process is precisely modulated by inhibitors. Accumulating evidence suggests that both activation and negative regulation of this signaling is crucial to bone development and malformation pathogenesis (Fig. 1).

### JAK-STAT signaling and craniomaxillofacial bone development

Craniofacial bone development can be classified into three distinct osteogenic patterns: intramembranous ossification, endochondral ossification, and a combination of both.

Intramembranous ossification involves the condensation of mesenchymal stem cells(MSCs) that express various proteoglycans to promote cell adhesion.^44^ These cells subsequently differentiate directly into pre-osteoblasts at ossification centers, which further transform into osteoblasts that secrete extracellular matrix proteins and establish an osteogenic microenvironment.^45^ The cranium and most facial bones develop through this process.^46^ The JAK-STAT signaling plays a significant role in this osteogenic process. In osteoblasts, JAK1, JAK3, and STAT3 promote osteogenic differentiation by upregulating key factors such as Runt-related transcription factor 2 (Runx2) and osteopontin (Opn), while STAT1 and STAT6 have been demonstrated to inhibit osteogenesis.^47,48^ This dynamic balance between promotion and inhibition is crucial for ensuring proper anatomical structure and bone density in craniofacial bones. Notably, the JAK-STAT pathway also plays key regulatory roles in osteoclasts. In osteoclasts, JAK3/STAT1, STAT5, and STAT6 are involved in functional regulation (STAT3 shows no significant regulatory effect): IL-3-activated STAT5 can inhibit osteoclastogenesis by upregulating the expression of dual-specificity phosphatases (Dusp)1 and Dusp2, while JAK3/STAT1 and STAT6 promote osteoclast differentiation and bone resorption.^15,49,50^

Endochondral ossification involves the initial differentiation of condensed MSCs into chondrocytes, which subsequently transdifferentiate into osteoblasts, with the remaining chondrocytes eventually being cleared by osteoclasts.^51^ The cranial base and temporal bones develop through this process.^52^ In endochondral ossification, JAK2-STAT3 mediates osteogenic differentiation of MSCs, and inhibition of this pathway impedes the ossification process.^53^ STAT1 primarily participates in chondrogenesis, potentially regulated by FGFR to promote pre-hypertrophic chondrocyte differentiation, and the developmental arrest observed in STAT1 gain-of-function mutations is associated with this process.^54^ STAT5 and STAT6 mainly regulate osteoclastic resorption. STAT5, activated by IL-3, inhibits RANKL-induced osteoclast formation.^16^ STAT6 is regulated by RANKL-activated NFAM1, leading to increased phosphorylation levels that promote osteoclast differentiation and bone resorption.^49^

The mandible is a typical tissue that develop from both intramembranous ossification and endochondral ossification.^55^ It forms the condyle, which participates in the formation of the temporomandibular joint (TMJ), the only movable joint in the face.^56^ Condensed MSCs differentiate into chondrocytes to form bilateral rod-shaped Meckel’s cartilage. This structure develops bilaterally along the mandibular arch, with the distal segment undergoing endochondral ossification to form articular structures, the middle segment undergoing intramembranous ossification to form the mandibular body, and the intermediate region undergoing endochondral ossification to form the symphysis. By 12 weeks of development, the condylar cartilage rapidly expands to occupy most of the mandibular ramus and undergoes endochondral ossification, leaving only a small portion of cartilage covering the condylar head.^55^ Postnatal growth and development of the condyle continue to influence craniofacial morphology. However, there are few reports on the role and mechanism of the JAK-STAT pathway in mandibular development, particularly its effects on different osteogenic modes in various segments, and it remains unknown whether the JAK-STAT pathway exhibits heterogeneity in this context. Morimoto et al. showed that STAT1 participates in regulating endochondral ossification in the distal segment and condylar formation, and its activation by double-stranded RNA-dependent protein kinase (PKR) promotes chondrogenic differentiation.^57^ Pagni et al. found that STAT3 exhibits nuclear localization in articular disc cells during human embryonic development and participates in transcriptional regulation.^58^ Notably, STAT3 displays distinct nuclear-cytoplasmic co-expression patterns in chondrogenic layers and ossification zones, suggesting its dual regulatory role in both chondrogenic differentiation and osteogenic processes. However, the specific molecular mechanisms remain to be fully elucidated.

### JAK-STAT signaling and dento-periodontal complex development

**Fig. 2:**
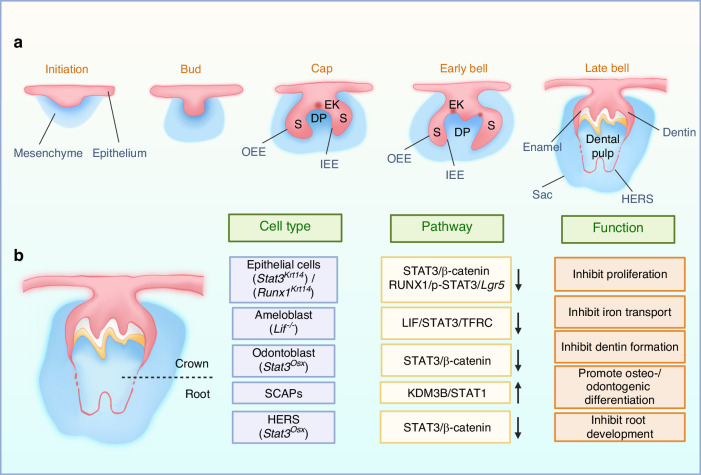
Changes in tooth development associated with JAK-STAT abnormalities. **a** Key stages of tooth development: bud stage, cap stage, and bell stage. These stages ultimately give rise to the enamel organ, dental papilla, and dental follicle, which correspond to the development of enamel, dentin-pulp complex, and periodontal tissues. **b** Functional abnormalities resulting from alterations in the JAK-STAT signaling in pivotal cells during development. Abbreviations: EK enamel knots, DP dental papilla, IEE inner enamel epithelium, OEE outer enamel epithelium, S stellate reticulum, HERS Hertwig’s epithelial root sheath (Created with bioRender.com)

During the eighth week of embryogenesis, epithelial cells invaginate into the embryonic mesenchyme. Around the ninth week, epithelial cells proliferate, with basal invagination forming a cap-like structure known as the enamel organ, which envelops the underlying dental papilla. In the next week, the enamel organ, dental papilla, and dental follicle become established, leading to the formation of enamel, the dentin-pulp complex, and periodontal tissues. The specific cells and key regulatory mechanisms are illustrated in Fig. 2.

Enamel formation consists of two phases: the secretory phase and the maturation phase. In the secretory phase, ameloblasts synthesize and secrete enamel matrix proteins. As they gradually transition into the maturation phase, ameloblasts enhance ion transport and pH regulation to thicken and widen enamel crystals. STAT3 has been shown to exert regulatory effects in both phases. Specifically, STAT3 is expressed in ameloblast progenitors and ameloblasts, where it promotes the synthesis and secretion of key enamel matrix proteins, including amelogenin, ameloblastin, and kallikrein-4.^19,59^ Furthermore, STAT3 is highly expressed in epithelial stem cells, orchestrates proliferation of epithelial cells and enhances enamel matrix protein expression through the *Runx1/Lgr5 axis*.^60^ Additionally, STAT3 regulates expression of the transferrin receptors, *Tfrc and Slc40a1*, modulating iron transport during the maturation phase, which affects the hardness and acid resistance of the incisor enamel.^61^

Epithelial-mesenchymal interactions are pivotal in tooth development, initiating the migration and aggregation of epithelial cells towards the mesenchyme in the bud stage and serving as a fundamental mechanism for root formation in the bell stage. In the bud stage, mesenchymal cells aggregate around the bud-shaped epithelium, expressing proteins that regulate the establishment of tooth-specific morphology. The dynamics of mesenchymal cells surrounding the epithelium are regulated by STAT1. Downregulation of STAT1 within the mesenchyme enhances its migratory capacity.^62^ Following the initial formation of the crown, the epithelium extends to form Hertwig’s epithelial root sheath (HERS), which interacts with the dental papilla mesenchyme to induce odontoblastic differentiation. The temporal coordination of these interactions significantly influences the length and morphology of the tooth root. Chan et al. indicated that STAT3 within mesenchymal cells may respond to signals from HERS via upregulation of Wnt/β-catenin signaling, thereby ensuring proper root elongation and formation of dentin.^18^ Concurrently, STAT3 also influences enamel formation indirectly but the specific mechanisms remain to be fully elucidated.

## The JAK-STAT signaling in dento-maxillofacial abnormalities

An increasing body of literature highlights the importance of the JAK-STAT signaling in the development, homeostasis, and repair processes of the skeletal system, particularly in relation to the emergence of dento-maxillofacial abnormalities. Reports indicate that mutation of STAT1, STAT3, STAT5A, and STAT5B induce various syndromes characterized by severe dentofacial deformities (Table 1), and corresponding mouse disease models have been developed to simulate the phenotypes observed in clinical patients (Supplementary Table 1). Research on JAK1 and JAK2 has primarily focused on bone marrow fibrosis and various inflammatory diseases, such as rheumatoid arthritis. While STAT6 was previously considered to have minimal significance for the skeleton, recent reports of STAT6 mutations leading to craniofacial deformities, as well as studies on its role in bone development, have emerged. Conversely, there are few reports on the roles of JAK3, TYK2, STAT2, and STAT4 in the skeletal system, suggesting that their contributions to dento-maxillofacial abnormalities may be limited.

**Table 1:** Abnormalities caused by mutation of JAK-STAT signaling

Gene	Disorder	Inheritance	OMIM number	Effects on JAK-STAT signaling	Phenotype	Reference
STAT3	Hyper-IgE Syndrome 1(HIES)	AD	147060	STAT3 dimerization decreased in all cells	Facial asymmetry, prominent forehead, mild mandibular prognathism, high palatal arch, and retention of deciduous teeth.	^21,101,102^
	STAT3 Gain-of-function Syndrome	AD	615952	STAT3 phosphorylation increased in all cells	Prominent forehead, characteristic round facies with cupped ears, dental anomalies.	^23,104,105^
STAT5B	Growth Hormone Insensitivity Syndrome with Immune Dysregulation1	AR	245590	STAT5B truncated and inactive in all cells	Prominent forehead, depressed nasal bridge.	^24^
	Growth Hormone Insensitivity Syndrome with Immune Dysregulation 2	AD	618985	STAT5B unable to nuclear localize or bind to DNA elements	Microcephaly	^25^
STAT6	Hyper-IgE Syndrome 6	AD	620532	STAT6 phosphorylation increased in all cells	Mild high palate arch, joint hyperextensibility, enamel hypoplasia, normal dentition without delay shedding in decidual teeth, severe growth retardation	^26,27^

### JAK1 in dento-craniofacial abnormalities

JAK1 is implicated in skeletal development and homeostasis. Individuals with heterozygous mutations in *JAK1*, primarily within the pseudokinase domain, exhibit gain-of-function (GOF) phenotypes, including excessive keratinization of the skin, autoinflammatory conditions, immune dysregulation, bone marrow fibrosis, and myeloproliferative disorders, but there is a lack of reported craniofacial phenotypes.^63,64^ In murine models, JAK1 deficiency results in perinatal lethality.^65^ Transgenic mice engineered to carry mutations that mimic those found in humans, such as H595D and S645P, show hyperactive *Jak1*, leading to reduced chondrocyte proliferation in the growth plates of long bones and decreased bone density.^66,67^ This effect appears to be independent of gender and extracellular phosphate levels.^68^ Notably, a study on cranial bone metabolism showed that mouse calvaria-derived osteoblasts, when stimulated by 1,25-dihydroxyvitamin D3 and prostaglandin E2, can induce the differentiation of osteoclasts. The mechanism involves JAK1 activation in osteoblasts, which mediates RANKL expression and thereby stimulates osteoclast formation.^69^

### JAK2 in dento-craniofacial abnormalities

In humans, mutations in *JAK2*, particularly the V617F somatic GOF mutation, are primarily linked to conditions such as polycythemia vera and essential thrombocythemia, as well as playing a significant role in the pathogenesis of myeloproliferative neoplasms.^70–72^ However, there are no documented cases of dento-craniofacial abnormalities directly resulting from *JAK2* mutations. Studies of murine models have revealed that *Jak2* knockout mice are unresponsive to erythropoietin, thrombopoietin, and IL-6, resulting in perinatal lethality due to impaired erythropoiesis.^73^ Notably, JAK2 in skeletal development appears to be a double-edged sword. Dodington et al. found that *Ctsk*^*Cre*^; *Jak2*^*fl/fl*^ mice demonstrate reduced body weight and shorter femur length compared with wild-type mice, though this phenotype does not correlate with typical osteoclastic resorptive activity. Downregulation of *Igf1* in these mice suggests that JAK2 may influence bone development through IGF1.^74^ Inhibition of JAK2 in bone marrow stromal cells has been shown to reduce the expression of osteogenesis-related genes and impair osteogenic function. Elevated levels of IL-6 and IL-6R in serum have been noted in patients undergoing orthognathic surgery, indicating a potential link to bone remodeling. In vitro, IL-6 stimulation of the osteocyte-like cell line MLO-Y4 leads to increased RANKL via p-JAK2 and promotion of osteoclastogenesis. Conversely, treatment with the JAK2 inhibitor AG490 effectively suppresses osteoclastogenesis.^75^ In osteoporosis, particularly in conditions of diabetes or menopause, JAK2 appears to have detrimental effects. Postmenopausal rats show downregulation of miR-211-5p alongside activation of the JAK2/STAT3 signaling, contributing to osteoporosis. Interestingly, upregulation of this micro RNA can target JAK2 to help mitigate bone loss.^76^

Studies of JAK2 in the dental-periodontal complex indicate that its activation has complex effects across different cell types. Zhu et al. simulated the hypoxic microenvironment during orthodontic tooth movement (OTM) in vitro and observed a significant enhancement in osteoclastogenesis when MLO-Y4 cells were co-cultured with osteoclast cells (RAW264.7). This process was mediated by HIF-1α-induced activation of the JAK2/STAT3 signaling, which stimulated osteoblastic RANKL expression, thereby indirectly regulating osteoclast differentiation. The mechanism may underlie how osteocytes modulate osteoclast-mediated OTM.^77^ In inflammation induced by nicotine and lipopolysaccharide in human periodontal ligament cells (PDLCs), inhibition of HIF-2α has been shown to block osteoclast differentiation by suppressing JAK2/STAT3 signaling.^78^ This suggests that JAK2 plays a dual role, promoting osteoclastogenesis in some contexts while inhibiting it in others. Jin et al. highlight that orthodontic force-induced tension activates JAK2/STAT3 signaling in PDGFRβ^+^ fibroblasts, leading to enhancement of new bone formation.^79^ This mechanism may be crucial for understanding how mechanical forces during orthodontic treatment influence bone remodeling. Moreover, maternal dietary intake during pregnancy, particularly regarding fatty acids, may influence fetal tooth development. Research has shown that administering trans-fatty acids or polyunsaturated fatty acids to pregnant rats results in significant differences in JAK2 expression, although no clear abnormalities in fetal dental tissues were observed. This raises interesting questions about the impact of maternal nutrition on the development of craniofacial structures via pathways involving JAK2.^80^

### JAK3 and TYK2 in dento-craniofacial abnormalities

JAK3 and TYK2, as members of the JAK family, are expressed in osteoblasts and osteoclasts. However, their contributions to craniofacial bone are not well-documented. *JAK3* deficiency is known to cause severe combined immunodeficiency (SCID), which is characterized by a lack of functional T cells and increased susceptibility to infections.^81^ There is a notable lack of reports regarding associated skeletal abnormalities in affected individuals. Mechanistically, JAK3/STAT3 signaling can be activated in osteoblasts, promoting the expression of osteogenic genes and enhancing mineralization processes.^48^ This suggests that JAK3 may be crucial for normal bone formation. In contrast, in osteoclasts, downregulation of the interferon (IFN)-β pathway appears to promote osteoclastogenesis. This may occur through the upregulation of long non-coding RNAs (lncRNAs) that interact with JAK3, ultimately leading to increased expression of Ctsk via the modulation of Nuclear factor of activated T-cells (*Nfatc1*).^82^ This highlights a potential dual role of JAK3 in balancing osteoblast and osteoclast activity, which is essential for maintaining bone homeostasis.

*TYK2* deficiency leads to autosomal recessive hyper-IgE syndrome (AR-HIES) marked by severe eczema and heightened vulnerability to infections.^83^ Notably, despite these immune challenges, there are no distinct skeletal manifestations. Animal studies support this observation, showing that *Tyk2*-deficient mice develop normally without evident skeletal anomalies.^84^ Li et al. indicate that IL-12 may enhance osteogenic differentiation through IL-12 receptor β1 (IL-12Rβ1)/TYK2/STAT3 signaling, particularly during radiation recovery.^85^ In Mucopolysaccharidosis VII (MPS VII), a disease with glycosaminoglycan accumulation and shortened long bones, levels of p-STAT3, TYK2, JAK1, JAK2 and leukemia inhibitory factor (LIF) decrease, leading to reduced chondrocyte proliferation in the growth plate.^86^ While JAK3 and TYK2 might play indirect roles in regulating bone homeostasis, their specific contributions to dento-maxillofacial abnormalities remain largely unexplored.

### STAT1 in dento-craniofacial abnormalities

GOF mutations in *STAT1* have been linked to growth retardation. A notable case involving a p.Ser466Arg alteration in *STAT1* revealed that the patient’s height and weight fell below the 5th percentile by age 15. This mutation site is located within the DNA-binding domain of STAT1, and the amino acid substitution exhibits an activating effect. The hyperactivation of interferon signaling pathways may represent the underlying cause of skeletal abnormalities.^28^ Patients with *STAT1* loss-of-function (LOF) mutations exhibit heightened vulnerability to severe infections during infancy. This is primarily attributed to impaired immune function, including the inability of interferons to activate STAT1.^87,88^ However, there is a lack of documented skeletal abnormalities associated with *STAT1* LOF mutations. Murine models further support this observation. Homozygous *Stat1* knockout mice (*Stat1*^−/−^) display no significant developmental defects in tissue and organ structures.^89^ *Stat1*^−/−^ mice exhibited increased bone mineral density accompanied by enhanced osteoclastogenesis and osteoblastogenesis. The augmented osteoblast differentiation resulted from the nuclear translocation of RUNX2, which normally sequestered in the cytoplasm by STAT1. Notably, this regulatory function of STAT1 operates independently of its phosphorylation activation, as it does not require the canonical phosphorylation site Tyr701 that is essential for STAT1’s transcriptional activity.^14^ Further investigations into STAT1 in intramembranous ossification were conducted using skull specimens from 14-week-old *Stat1*^−/−^ mice. These specimens revealed a 28% increase in skull thickness and an elevated count of osteoclasts compared to littermates. Mechanistically, the findings suggest a complex regulatory interplay involving STAT1, characterized by decreased expression of the cell cycle inhibitor p21WAF/CIP and fibroblast growth factor receptor 3 (FGFR3), alongside increased expression of *Fgf18*.

STAT1 plays a vital role in inflammation within the dento-periodontal complex, especially during OTM. Under mechanical stimulation, hydrogen sulfide produced by periodontal ligament stem cells (PDLSCs) promotes bone remodeling and tooth movement by activating STAT1 in macrophages to drive M1 polarization.^90^ In periodontitis, the downregulation of STAT1 and Suppressor of cytokine signaling (SOCS)1 and SOCS3 in PDLCs leads to increased levels of pro-inflammatory cytokines such as IL-6 and IL-8, creating an inflammatory environment.^91^ This inflammatory state was further exacerbated because of reduced cell proliferation and impaired osteogenic differentiation, mediated by Zinc finger E-box-binding homeobox (ZEB)1 regulation of 5’-AMP-activated protein kinase (AMPK)/ Rho-associated protein kinase (ROCK)1 and its downstream effects on B-cell lymphoma (BCL)6/STAT1.^92^ Zhang et al. found that *Mir338*^*-/-*^ mice exhibited diminished polarization of macrophages toward the M1 phenotype and reduced osteoclastogenesis, again mediated through STAT1 pathways.^93^ Beyond its influence on osteoblasts and osteoclasts, STAT1 also affects the proliferation and differentiation of other critical cell types in the dento-periodontal complex. In stem cells from the apical papilla (SCAPs), histone demethylase KDM3B enhances osteo- and odontogenic differentiation, along with cell proliferation and migration, via STAT1 activation.^94^ Dernowsek et al. revealed that the induction of human exfoliated deciduous teeth (SHED cells) toward osteoblastic differentiation resulted in a gradual downregulation of STAT1 mRNA while upregulating RUNX2.^17^

### STAT3 in dento-craniofacial abnormalities

Research on dento-maxillofacial abnormalities associated with *STAT3* mutations in humans is notably prominent compared to other members of the STAT family. This heightened attention is largely attributed to Job’s syndrome, which arises from dominant-negative *STAT3* mutations and accounts for approximately two-thirds of all cases of this condition.^22,95^ First reported in 1966, Job’s syndrome is classified as a primary immunodeficiency disorder characterized by distinct clinical manifestations, including specific dermatitis, recurrent skin infections primarily due to *Staphylococcus aureus*, and frequent pulmonary infections.^96–98^ Elevated plasma immunoglobulin E (IgE) levels observed in early childhood leads to the alternative designation of hyper-IgE syndrome. Job’s syndrome can be divided into two types: AD-HIES and AR-HIES. AD-HIES is characterized by systemic symptoms include musculoskeletal abnormalities, while AR-HIES typically lacks skeletal manifestations.^99,100^ Our discussion primarily focuses on AD-HIES, where patients may present with craniosynostosis at birth. Distinctive facial features, such as facial asymmetry, a prominent forehead, a broad nasal bridge, prognathism, and a high-arched palate, may develop during adolescence or even earlier. In terms of dental anomalies, reduced absorption of deciduous tooth roots can lead to the retention of multiple primary teeth in affected individuals. Moreover, varying degrees of skeletal abnormalities can be observed, including reduced bone density and increased susceptibility to pathological fractures of long bones and ribs. Some patients may also exhibit scoliosis and joint hyperextensibility.^21,101,102^ In a cohort study focusing on HIES and chronic mucocutaneous candidiasis, skeletal or dental abnormalities were observed in 46.2% of the patients. The most frequently reported feature was a characteristic facial appearance (23.1%), followed by retained primary teeth, which was reported in 18.9% of the patients.^103^

*STAT3* gain-of-function (GOF) mutations give rise to a condition known as *STAT3* GOF syndrome, which is characterized by distinctive facial phenotypes including a rounded visage, prominent forehead, elongated upper lip, and smooth philtrum. Cup-shaped ears are also a prominent feature, and these facial characteristics exhibit consistency across age, gender, and ethnic backgrounds.^23^ In addition to the notable facial dysmorphia, patients with *STAT3* GOF syndrome present with systemic skeletal anomalies, which may include arthritis and stunted stature.^104^ Documented cases have reported bilateral underdevelopment of the first rib, highlighting the skeletal manifestations associated with this condition.^105^ A hallmark of *STAT3* GOF *s*yndrome is immune dysregulation, evidenced by lymphoproliferation and a decrease in various autoimmune cells. This dysregulation impacts multiple visceral organs, including the liver, intestines, lungs, and kidneys, and is associated with an increased incidence of malignancies relative to the general population.^106,107^ STAT3 GOF mutations are distributed across all domains of the protein. Notably, mutations within the DNA-binding domain induce a constitutively activated state characterized by delayed dephosphorylation, while the pathogenic mechanisms of variants in other domains remain to be fully elucidated. Emerging evidence suggests that STAT3 GOF may impair the terminal exhaustion of CD8 + T cells, thereby contributing to immune dysregulation.^106^ Current research efforts predominantly focus on the immunological aspects of *STAT3* GOF syndrome, with limited attention given to the intricate facial features that characterize affected individuals.

In the context of *STAT3* dominant-negative mutations associated with Job’s syndrome, research utilizing mouse models typically involves the knockout or knockdown of *Stat3* within the murine system. Zhou et al. demonstrated that deletion of *Stat3* in osteoblasts within *Osx*^*Cre*^;*Stat3*^*fl/fl*^ mice resulted in skeletal defects that resemble the features of AD-HIES. These defects include craniofacial dysmorphia, osteoporosis, and spontaneous bone fractures, while deletion of *Stat3* in osteoclasts did not produce any notable phenotypic changes.^108^ Additionally, Yadav et al. indicated that the deletion of *Stat3* in limb mesenchymal cells or osteoprogenitor cells during mouse development, in *Prrx1*^*Cre*^;*Stat3*^*fl/fl*^ mice, resulted in limb shortening, bending, and multiple long bone fractures. Notably, these alterations occurred without affecting chondrocyte hypertrophy and differentiation.^109^ In addition, Huang et al. highlighted the presence of open cranial fontanels in *Prrx1*^*Cre*^;*Stat3*^*fl/fl*^ mice.^110^ Mechanistic investigations suggest that the deletion or reduced activity of STAT3 is associated with decreased transcription levels of *Distal-less homeobox (Dlx)5* and diminished Wnt/β-catenin signaling, both of which are crucial for craniofacial skeletal development.^108,109^ Furthermore, research on monocytes from patients with HIES and monocytes subjected to STAT3 inhibition indicated that under IL-6 stimulation, both groups with inhibited STAT3 exhibited upregulation of OPN compared to normal individuals and cells not treated with inhibitors.^111^ This finding suggests that STAT3 may also play a role in the formation of facial dysmorphia in HIES by responding to IL-6 and promoting the expression of OPN, thereby contributing to the observed craniofacial abnormalities.^111^ Further reports on STAT3 have focused on its role in long bones. In 18-week-old osteocyte-specific STAT3 knockout mice, *Dmp1*^*Cre*^;*Stat3*^*fl/fl*^ mice, no significant differences were observed in femoral bone density, trabecular bone, or cortical bone. However, osteoblast formation was reduced, and osteoclast formation was increased, with a decreased bone formation response to mechanical stress observed in vitro.^112^ *Col1α1*^*Cre*^;*Stat3*^*fl/fl*^ mice exhibited osteoporosis due to a reduced bone formation rate.^113^ In osteoclast-specific STAT3 knockout mice, *Ctsk*^*Cre*^;*Stat3*^*fl/fl*^ mice, analysis of femoral bones at 8 weeks revealed that female mice experienced more severe bone loss compared to male mice, which is associated with estrogen receptor regulation.^114^ SOCS3, a key inhibitor of STAT3, was found to delay both the reduction in cortical porosity and the transition from low to high-density bone in *Dmp1*^*Cre*^;*Socs3*^*fl /fl*^ mice. Additionally, STAT3 phosphorylation in response to gp130-signaling cytokines was prolonged, while responses to Granulocyte Colony-Stimulating Factor (G-CSF) or leptin remained unaffected.^115^

In the dento-periodontal complex, STAT3 functions as a crucial mediator in response to mechanical stress, cytokines, and other external stimuli. In *Osx*^*Cre*^;*Stat3*^*fl/fl*^ mice, notable features include shortened roots of molars and incisors, along with thin dentin; similar defects are also observed in mandibular incisors. Histological analysis of the dental pulp matrix in these conditional knockout (CKO) mice reveals a significant reduction in Ki67^+^ proliferating cells, indicating impaired cellular proliferation.^18^ Moreover, a decrease in β-catenin signaling is detected in the HERS and dentin cells, reinforcing the role of STAT3 in craniofacial skeletal abnormalities through the Wnt/β-catenin signaling pathway.^18^ In addition, *Stat3* deletion in epithelial cells involved in enamel formation—in *Krt14*^*Cre*^;*Stat3*^*fl/fl*^ mutant mice—results in minimal enamel mineralization observed in the incisors beneath the mesial root of the first molar. Interestingly, the erupted portion shows a comparable microscopic structure of enamel mineralization, suggesting that the absence of STAT3 in epithelial cells delays the enamel formation process specifically in incisors.^19^ Further investigations into osteoblast function reveal that inactivation of STAT3 slows the rate of OTM and reduces both bone resorption and formation. *Stat3*-deficient osteoblasts also exhibit an inhibitory effect on osteoclast formation through interactions between osteoblasts and osteoclasts. This inhibition may involve binding of STAT3 to the *matrix metalloproteinase (Mmp)3* promoter, thereby regulating its transcriptional activity, which is vital for osteoclast differentiation and function.^116^

STAT3 plays a pivotal role in the regulation of various molecules within the dento-periodontal complex, particularly during the differentiation processes of ameloblasts and odontoblasts. Evaluation of 20 male and female human embryos indicates that the expression of *Stat3*, along with *Octamer-binding transcription factor (Oct)4*, *Nanog*, and *SRY-related HMG-box gene (Sox)2*, is closely associated with these differentiation processes.^59^ Immunostaining for STAT3 at the bell stage reveals its localization in both the nuclei and cytoplasm of cells across all regions.^59^ More targeted studies utilizing CKO mice, specifically *K14*^*Cre*^;*Runx1*^*fl/fl*^ mice with inactivated *Runx1*, demonstrate significant developmental defects, including markedly shortened incisors, underdeveloped cervical loops, and enamel defects. Within the cervical loop of these mutants, cell proliferation in dental epithelial cells is notably impaired, alongside downregulation of *Leucine-rich repeat-containing receptor (Lgr)5* and enamel matrix proteins, which correlates with decreased STAT3 phosphorylation levels.^60^ Fan et al. revealed that *Lif*^*-/-*^ mice had shorter incisors with reduced enamel surface hardness and acid resistance. This is attributed to diminished expression of key iron transport proteins, *Tfrc* and *Slc40a1*, in incisor progenitor cells, mediated by *Lif* through activation of STAT3 signaling.^61^ Additionally, stem cells derived from human SHED cells demonstrate the capacity to differentiate into vascular endothelial cells and odontoblast-like cells. In vitro studies show that vascular endothelial growth factor (VEGF) induces SHED cell expression of endothelial markers such as VEGFR2, CD31, and VE-Cadherin, facilitating the formation of capillary-like structures. Notably, this process is accompanied by inhibition of STAT3 phosphorylation, highlighting a relationship between STAT3 activity and cell stemness.^61^ STAT3 also responds to mechanical forces during OTM, acting as a potential mediator of mechanotransduction. The insertion of a nickel-titanium spring between the maxillary left lateral incisor and the first molar enhances the expression of STAT3, IL-6, gp130, and small heterodimer partner (SHP)2 mRNA on both tension and compression sides compared to controls.^117^ Mechanistic studies indicate that, on the tension side, Platelet-derived growth factor-BB (PDGF-BB) promotes new bone formation via activation of the JAK2/STAT3 signaling in PDGFRβ^+^ fibroblasts.^79^ In the context of inflammation, surgical cortical bone osteotomy induces M1 polarization in macrophages through NF-κB pathway activation, subsequently leading to M2 polarization via JAK/STAT3 signaling. This transition triggers the release of inflammatory cytokines and enhances bone resorption, thus contributing to an increased rate of OTM locally.^118^

### STAT5 in dento-craniofacial abnormalities

STAT5 comprises two isoforms, STAT5A and STAT5B, encoded by different genes. While these isoforms exhibit high sequence homology and structural similarity, they differ in their expression patterns and functional roles within the human body.^119^ Patients with LOF mutations in *STAT5B* experience severe developmental delays and exhibit a significant lag in bone age compared to chronological age.^24,120^ Interestingly, Klammt et al. reported a dominant-negative *STAT5B* mutation in which most patients exhibited short stature and delayed bone age, with some patients showing microcephaly. This mutation allows STAT5B to be phosphorylated and activated but prevents its translocation to the nucleus or binding to DNA elements. Additionally, the abnormal STAT5B can dimerize with normal STAT5B, thereby interfering with the normal function of wild-type STAT5B.^25^ Conversely, those with GOF mutations in *STAT5B* may present with eosinophilia, which progresses to chronic eosinophilic leukemia; however, their skeletons and development typically remain normal.^121^ In a case involving a patient with a *STAT5A* mutation in conjunction with SH2B3, activation of the p-STAT5A/c-Myc/Cyclin D1 and p-STAT3/p-AKT/p-ERK1/2 signaling axes was observed, which promotes tumorigenesis and is accompanied by myelofibrosis.^122^ In murine models, *Stat5a* and *Stat5b* double knockout (*Stat5a*^*−/−*^*5b*^*−/−*^) mice demonstrate severe anemia during embryonic development, with survival analyses indicating early mortality.^123^ These mice exhibit a reduction in erythroid progenitor cells, an increase in apoptosis, and a decrease in CD4^+^CD25^+^ regulatory T cells within the bone marrow.^123^ Research has indicated that the absence of STAT5A, rather than STAT5B, promotes osteogenesis in human bone marrow stromal cells (hBMSCs). The underlying mechanism appears to involve STAT5A negatively regulating the transcription factor *Dlx5*.^124^ Studies on *Stat5a*-deficient mice have revealed increased vertebral and cortical bone mass, enhanced bone mineral density, prevention of bone loss, and promotion of fracture healing.^124^ Mice with osteoclast-specific knockout of *Stat5* (*Ctsk*^*Cre*^;*Stat5*^*fl/fl*^) exhibit an osteoporotic phenotype characterized by enhanced osteoclast resorptive capacity.^15^ Mechanistically, IL-3 activates STAT5, which subsequently inhibits osteoclast bone resorption by upregulating the expression of the dual-specificity phosphatases *Dusp1* and *Dusp2*.^15^*Mx1*^*Cre*^;*Stat5*^*fl/fl*^ mice exhibited an increased number of osteoclasts and decreased bone mass. STAT5 inhibits RANKL-induced osteoclast formation, but STAT5 itself is not influenced by RANKL. Additionally, STAT5 is activated by IL-3, promoting the expression of *Inhibitor of DNA binding(Id)2* and *Id3*, thereby suppressing osteoclast activity.^16^ In adipocyte-specific *Stat5* knockout mice (*Apn*^*Cre*^;*Stat5*^*fl/fl*^), enhanced adipogenic differentiation of BMSCs is observed, alongside a reduction in bone mass. This reduction in bone mass may be attributed to STAT5 binding to the promoter of *Activating transcription factor (Atf)3*, positively regulating its expression. Overexpression of *Atf3* rescues the increased adipogenesis induced by STAT5 knockout.^125^ Additionally, co-culturing macrophages with adipocytes from *Stat5* CKO mice promotes macrophage differentiation into osteoclasts, potentially contributing to the observed reduction in bone mass.^125^

While numerous studies have underscored the positive regulatory role of STAT5 in osteogenesis within long bones, there is a notable lack of research specifically addressing its potential role in the mandible and facial tissues. This gap suggests a promising avenue for future investigation, as understanding the involvement of STAT5 in craniofacial bone development could yield valuable insights into the mechanisms underlying craniofacial abnormalities and contribute to the development of targeted therapeutic strategies.

### STAT6 in dento-craniofacial abnormalities

Historically, STAT6 was not regarded as significantly impacting the skeletal system; however, recent studies have begun to illuminate its important role in dento-craniofacial abnormalities. Baris et al. reported a case of a 10-year-old boy with a novel heterozygous G > A substitution in exon 22 of *STAT6*. An examination at age 8 showed various craniofacial features, including rough facial soft tissue, a widened nasal bridge, enlarged nasal alae, deep eye sockets, a slightly heightened palatal arch, joint hyperextensibility, enamel hypoplasia, and normal dentition without delayed shedding of deciduous teeth.^26^ The mutation results in elongation of the STAT6 side chain and an increased DNA interaction without affecting the dephosphorylation kinetics, causing severe growth retardation.^26^ Another study indicated that short stature is a common characteristic in patients with *STAT6* GOF mutations, with 5 out of 16 surveyed individuals presenting with additional skeletal issues, such as pathological fractures and generalized hypermobility.^27^ Beyond skeletal manifestations, the most frequently reported clinical symptoms associated with *STAT6* mutations include atopic dermatitis and food allergies, accompanied by increased eosinophil counts and significantly elevated serum IgE levels.^27^ Analyses revealed that STAT6 GOF mutations drive enhanced Th2 cell responses coupled with suppressed Th1 responses, resulting in immune dysregulation.^27^ Research on animal models primarily focused on the effects of *Stat6* knockdown or knockout on the skin, liver, and immune system, with limited investigation into the mechanisms underlying potential bone-related diseases.^126–128^ Studies on the role of STAT6 in osteoclasts and osteoblasts reveal potential mechanisms underlying craniofacial bone development and homeostasis. Stimulation with IL-4 or IL-13 rapidly phosphorylates STAT6, which in turn inhibits the phosphorylation and nuclear translocation of nuclear factor kappa-B (NF-κB), diminishing its DNA-binding ability and suppressing osteoclastogenesis.^129,130^ Notably, IL-13 is recognized as a factor that inhibits bone resorption.^131^ In vitro studies involving osteoblasts isolated from mouse calvaria demonstrated that treatment with IL-4 or IL-13 downregulated receptor activator of NF-κB (RANK) and upregulated osteoprotegerin (OPG). These effects were not observed in the calvaria of *Stat6* knockout mice, while they were evident in wild-type and *Stat4* knockout mice.^131^ Additionally, Ethiraj et al. indicated that receptor activator of nuclear factor kappa-B ligand (RANKL) promotes osteoclast differentiation and bone resorption by upregulating STAT6 phosphorylation via *Nfam1* expression, a pathway seemingly distinct from STAT3 involvement.^49^ During long bone repair, activation of the JAK1/STAT6 signaling in macrophages promote M2 polarization, enhancing angiogenesis and bone regeneration.^132^

Aggressive periodontitis (AgP) is a disease that primarily affects healthy young individuals, characterized by the rapid destruction of periodontal tissues. Compared to control groups, patients with AgP exhibit an increased frequency of specific IL-4 gene variants, as well as elevated expression of IL-4 and STAT6 in CD4^+^ T cells. These findings suggest a potential association between STAT6 overexpression and the pathogenesis of AgP.^133^ Notably, deficiency in STAT6 has been shown to confer resistance to alveolar bone loss induced by *Tannerella forsythia*, a key pathogen in periodontal disease.^134^ In summary, the dento-maxillofacial abnormalities observed in patients with reported STAT6 mutations further underscore the significance of this transcription factor in craniofacial development and health. However, the specific pathogenic mechanisms underlying these anomalies remain unclear, and the key cellular subpopulations in which STAT6 primarily functions are yet to be identified.

### STAT2 and STAT4 in dento-craniofacial abnormalities

Research into the role of STAT2 in craniofacial bones and even the skeletal system is limited. Homozygous mutations in *STAT2* have been linked to severe immunodeficiency disorders and heightened susceptibility to viral infections.^135^ Studies utilizing *Stat2*-deficient (*Stat2*^*−/−*^) mice have demonstrated defects in immune responses and an increased vulnerability to viral infections.^136^ *STAT2* mutations may not directly cause dentofacial developmental abnormalities, but its overactivation is involved in the regulation of cranial bone development. In Paget’s disease of bone, which is characterized by abnormal cortical bone resorption in long bones and specific alterations in the skull, research has revealed early destruction of the outer table of the skull while preserving the inner table, accompanied by abnormal thickening of the skull cap and often leading to depression of the skull base.^137,138^ A study investigating peripheral blood mononuclear cells (PBMCs) from patients found upregulation of both STAT2 and STAT1 compared to control groups, suggesting a potential involvement of the STAT2 and STAT1 signaling pathways in abnormal osteoclast formation and bone resorption.^138^ However, the precise mechanisms underlying these observations necessitate further investigation.

Similar to STAT2, abnormalities in STAT4 appear to have minimal impact on craniofacial development. A 2023 report from NEJM identified a GOF mutation in *STAT4* that leads to a systemic inflammatory disorder known as Disabling Pansclerotic Morphea (DPM), characterized by skeletal developmental abnormalities and joint stiffness. Although this report did not mention craniofacial bones, it suggests that future studies should investigate whether DPM patients exhibit facial dysmorphology or temporomandibular joint disorders.^29^ *Stat4*-deficient (*Stat4*^*-/-*^) mice are viable and fertile; however, they display an impaired response to IL-12-mediated signaling, resulting in inhibited T helper 1 (Th1) activation and subsequent immune system abnormalities.^139,140^ Notably, these mice do not show significant differences in body size compared to their wild-type counterparts.^139^ In research related to osteoporosis, analysis of data from the Gene Expression Omnibus database, alongside experiments using human blood samples, highlighted the involvement of the Palbociclib/miR-141-3p/STAT4 axis. This axis targets and degrades STAT4 mRNA via miR-141-3p, contributing to alleviation of the oxidative stress associated with osteoporosis.^141^ These findings suggest that heightened abnormalities in STAT4 could potentially disrupt the homeostasis of the dento-periodontal complex, indicating a need for further investigation into its role in craniofacial and periodontal health.

### Inhibitor of JAK/STAT signaling in dento-craniofacial abnormalities

The classic negative regulators of the JAK/STAT signaling include the SOCS family, Protein inhibitor of activated STAT (PIAS) family, and protein tyrosine phosphatase (PTP). Activation of the JAK/STAT signaling leads to increased transcription of SOCS, which inhibits STAT phosphorylation by binding to JAK.^142^ SOCS2 inhibits excessive body growth and negatively regulates growth hormone (GH) signaling. *Socs2*^*-/-*^ mice exhibit a 30-50% increase in body size and show jaw abnormalities, with an increased number of osteoclasts.^143,144^ Li et al. constructed *Socs2*^*R96C/R96C*^ mice, disrupting the binding of SOCS2 to phosphorylated tyrosine, which resulted in a phenotype similar to *Socs2*^*-/-*^ mice. Both knockout mouse strains exhibited cranial growth and a corresponding increase in overall skeletal size, due to excessive STAT5 phosphorylation following GH stimulation.^145^ SOCS3 primarily inhibits STAT3 phosphorylation, and its role in the cortical bone of long bones has been reported.^47^ Young male and female Dmp1^*Cre*^;*Socs3*^*f/f*^ mice show increased trabecular bone mass and abnormal cortical bone at the epiphyseal end. Adult male mice return to normal, while females remain abnormal, possibly due to androgens promoting cortical bone formation via IL-6.^115,146^ A recent study involved construction of *Fgfr3*^*N534K/+*^ hypochondroplasia (Hch) mice and *Fgfr2c*^*C342Y/+*^ Crouzon syndrome (Crz) mice, and observed mandibular development and the healing of unstable mandibular fractures. Crz mice exhibited abnormally high mineralization in fracture calluses, while Hch mice showed abnormally low mineralization and overexpression of *Socs3*.^147^ SOCS7 is significantly expressed in the brain, and *Socs7*^−/−^ mice are smaller than their littermates, prone to hydrocephalus, and develop cranial deformities leading to death.^148^ PIAS family proteins are constitutively expressed and inhibit STAT activity by binding to STAT, preventing STAT-DNA binding, and promoting STAT SUMOylation.^13^ There is currently a lack of reports on PIAS abnormalities causing dentofacial deformities. Ali et al. found that mechanical stress promotes osteoblast differentiation in mouse cranial bones, with PIASxβ transiently upregulated during this process and promoting osterix expression, possibly by affecting NFATc1 or NFATc3 SUMOylation.^149^ Additionally, a proteomic study of patients with degenerative scoliosis found high levels of PIAS2, although the specific mechanism remains unclear.^150^ PTPs are proteins that remove phosphate groups from phosphorylated tyrosine residues, affecting pathways such as JAK/STAT, Ras/MEK/ERK, and PI3K/Akt. Among them, SHP1 and SHP2 are associated with dento-maxillofacial abnormalities.^151,152^ Tang et al. found that SHP1 is highly expressed during differentiation and mineralization in cranial-derived osteoblasts of mouse embryos, promoting these processes by binding to GSK3β.^153^ SHP2 mutations may cause Noonan syndrome, characterized by the absence of cranial, nasal, and mandibular bones, primarily related to the Ras/MEK/ERK pathway.^51^

**Fig. 3:**
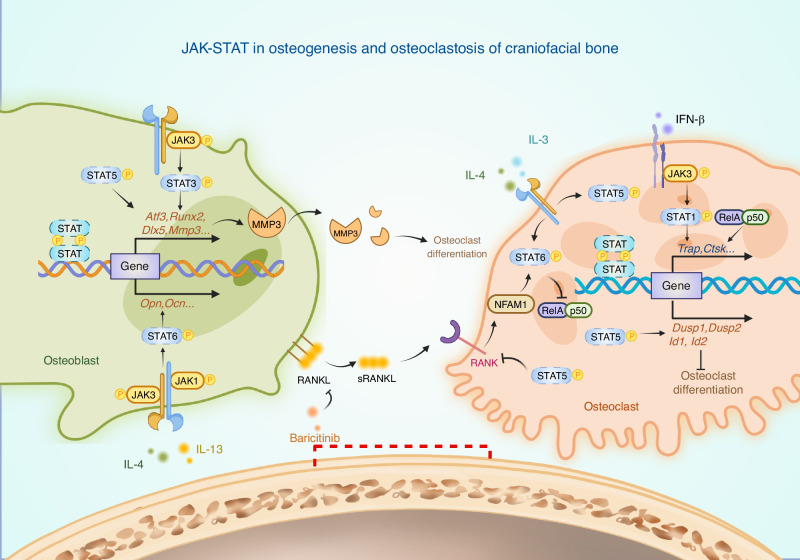
The JAK-STAT signaling plays a pivotal role in regulating the mechanisms underlying osteogenesis, osteoclastogenesis, and the crosstalk between them. Throughout the processes of craniofacial bone development, homeostasis, and repair, there is a continuous and active interplay between osteogenic and osteoclastic activities. Key regulatory molecules, including IL-3, IL-4, IL-13 and IFN-β, are integral to this complex regulatory network. Additionally, JAK1, JAK3, STAT3, STAT5, and STAT6 serve as essential transducers of extracellular signals, while RANKL and MMP3 act as critical mediators of extracellular crosstalk within this context (Created with bioRender.com)

**Fig. 4:**
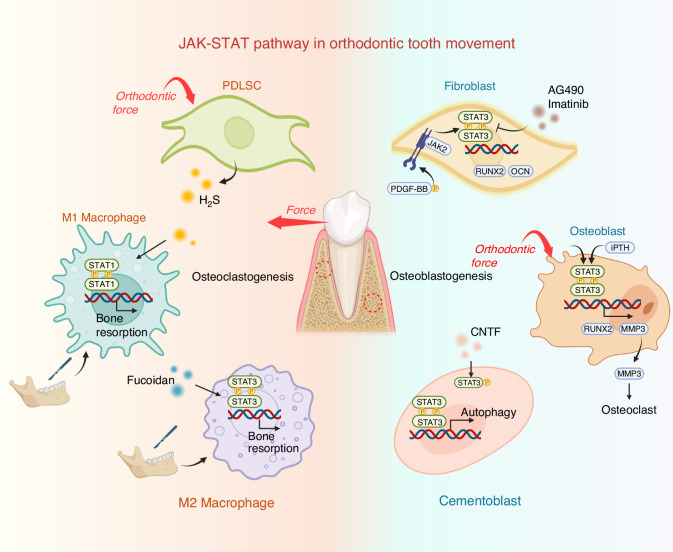
The JAK-STAT signaling and target molecules in orthodontic tooth movement. Orthodontic tooth movement is characterized by two pivotal processes: bone resorption and bone formation. Activation of STAT3 within fibroblasts and osteoblasts is critical for promoting new bone formation, while phosphorylated STAT3 in cementoblasts plays a crucial role in inducing autophagy and facilitating bone resorption. Additionally, hydrogen sulfide produced by periodontal ligament stem cells (PDLSCs) and macrophage activation of STAT1 in response to surgical corticotomy of the alveolar bone both promote polarization toward the M1 phenotype. This surgical intervention also triggers subsequent STAT3 activation, driving the transition to the M2 phenotype, thereby enhancing bone resorption. Notably, AG490, Imatinib, and ADPN function as inhibitors of STAT3, while Fucoidan, iPTH, and CNTF are known to promote STAT3 phosphorylation. Abbreviations: CNTF: ciliary neurotrophic factor; iPTH: intact parathyroid hormone; PDGF-BB: platelet-derived growth factor-BB; PDLSC: periodontal ligament stem cells (Created with bioRender.com)

In summary, STAT1, STAT3, STAT5A, and STAT5B within the JAK-STAT signaling and inhibitor family are integral to the occurrence and development of craniofacial bone deformities, primarily through their regulation of osteogenesis and osteoclastogenesis (Fig. 3). OTM is a critical clinical intervention for dentoalveolar complex abnormalities, with the STAT1 and STAT3 being identified as central mediators in mechanotransduction. This pathway orchestrates three fundamental biological processes: inflammatory response, pressure-side bone resorption, and tension-side bone formation, which are essential for alveolar bone remodeling in response to mechanical stimuli (Fig. 4).

## The JAK-STAT signaling in clinical treatment

Given the critical role of JAK-STAT signaling in the onset and progression of dento-maxillofacial abnormalities, targeting this signaling shows promise as a strategy for the treatment and prevention of deformities. Currently, therapeutic molecules aimed at this signaling can be classified into three primary categories: chemical and natural compounds, proteins, and non-coding RNAs. Functions and potential indications of these various molecules are systematically summarized in Supplementary Table 2.

### Chemical and natural compounds

Chemicals and natural compounds targeting JAK1 and JAK2 have emerged as significant inhibitors in the pharmacological landscape, with Ruxolitinib being the most widely recognized and FDA-approved medication. Ruxolitinib functions by competing with adenosine triphosphate (ATP) to inhibit the phosphorylation of both JAK1 and JAK2, thereby disrupting downstream signaling pathways.^154^ In addition to Ruxolitinib, several other compounds have attracted attention for their therapeutic potential.^155^ Notably, ruxolitinib demonstrates efficacy in suppressing calvarial bone resorption. In a murine model of implant-induced cranial osteolysis, the bone resorption process was accompanied by significant intraosseous lymphatic vessel expansion. Enhanced lymphangiogenesis was shown to effectively mitigate osteoclastic bone loss. Administration of ruxolitinib restored lymphatic vessel responsiveness to external stimuli in aged mice, suggesting its potential for preventing periprosthetic osteolysis during dentofacial deformity treatments.^156^ Preliminary evidence also indicates ruxolitinib may have therapeutic value in long bone loss, though further validation is required.^157^ Baricitinib, the second FDA-approved JAK1/JAK2 inhibitor, has demonstrated clinical benefits in improving osteoporosis and increasing bone mineral density Z-scores.^69,158^ However, its therapeutic effects on craniofacial bones remain to be elucidated. Other notable compounds, such as Tofacitinib, GLPG0634, and SHR0302, have demonstrated efficacy in mitigating cartilage loss and inflammation.^159–161^ Specifically, Tofacitinib, a selective inhibitor of JAK1 and JAK3, has been shown to inhibit dysfunctional osteoclast activity in rheumatoid arthritis, effectively reducing bone loss.^159,162^ GLPG0634, characterized as a selective JAK1 inhibitor, may contribute to reduced cartilage and bone degradation by inhibiting the differentiation of T helper cells (Th1, Th2, and Th17), thereby alleviating associated inflammatory responses.^161^ SHR0302 has also shown promise, as it alleviates the severity of adjuvant-induced arthritis in rat models by inhibiting JAK1/STAT3 phosphorylation and modulating Th17 cell function, alongside a reduction in the overall population of B cells.^160^ Furthermore, the natural compound Rhizoma Dioscoreae extract has been investigated for its protective effects against alveolar bone loss in ovariectomized rats. This extract appears to regulate JAK1/STAT3 signaling through microRNA mechanisms, suggesting a potential avenue for therapeutic intervention in bone loss conditions.^163,164^ The extract also exhibits comparable therapeutic effects in long bone osteoporosis, demonstrating similar efficacy.^165^

Fedratinib has also emerged as a noteworthy agent demonstrating positive effects on bone integrity.^166^ In vitro studies involving human bone marrow-derived stem cells (hBMSC) have shown that Fedratinib effectively inhibits JAK2, resulting in the decoupling of downstream signaling pathways such as STAT3, thereby obstructing the osteogenic differentiation of hBMSCs.^167^ Fedratinib has received FDA approval for the treatment of myelofibrosis and is particularly advantageous for patients exhibiting resistance to Ruxolitinib.^168^ Huang et al. found that 2,3,5,4’-tetrahydroxystilbene-2-O-beta-glucoside (THSG) enhanced the phosphorylation levels of JAK2 and STAT3, which in turn improved cell viability, telomerase activity, and the ability to form embryoid bodies in human dental pulp stem cells (DPSCs). This highlights the significant potential of THCG for alternative cell therapy strategies aimed at tooth regeneration and repair.^169^ In addition to direct inhibition, several compounds exert their effects through indirect mechanisms targeting JAK2. Metformin, a widely prescribed medication for type 2 diabetes, not only lowers blood glucose levels but also offers protective effects on bone health. Its mechanism may involve the reduction of phosphorylation levels of both JAK2 and STAT1, thereby promoting the osteogenic differentiation of stem cells.^170^ The traditional Chinese medicine extract Eupatilin has been shown to upregulate miR-211-5p, which targets and inhibits JAK2, subsequently suppressing STAT3 activation and thereby improving osteoporosis in postmenopausal rat models.^76^ Additionally, a bioactive mixture derived from Humulus japonicus has been reported to promote the phosphorylation of the JAK2-STAT5 signaling, facilitating insulin-like growth factor 1 (IGF-1) synthesis. This effect has been associated with enhanced growth parameters, including increased nasal and tail lengths, as well as improved femur and tibia lengths during the development of rats.^171^

Patients with STAT1 mutations do not exhibit significant abnormalities, but STAT1 does play a regulatory role in the dento-maxillofacial developmental process.^172^ Resveratrol, a polyphenolic compound with estrogen-like activity, demonstrates significant bone-protective effects in both animal and clinical studies, enhancing vertebral and femoral bone mineral density. Beyond its anti-osteoporotic properties, resveratrol also exhibits therapeutic potential against periodontitis. It not only inhibits STAT1 phosphorylation but also activates STAT3 phosphorylation, promoting the M2 polarization of macrophages and thereby alleviating periodontal inflammation.^173^ During orthodontic tooth movement, PDLSCs produce hydrogen sulfide, which upregulates the phosphorylation levels of STAT1 in macrophages and facilitates M1 polarization, resulting in an elevated number of osteoclasts and accelerating the rate of tooth movement.^90^ The potent STAT1 inhibitor fludarabine has been shown to upregulate *Osterix* expression, thereby promoting bone formation during fracture healing.^172^ Moreover, fludarabine inhibits the formation of empty lacunae in trabecular bone through the STAT1/Caspase-3 signaling axis, contributing to improved outcomes in cases of steroid-induced avascular necrosis of the femoral head (SANFH).^174^

The activation or inhibition of STAT3 in various cell types yield opposing effects on bone metabolism. Classic STAT3 inhibitors, such as AG490 and imatinib, have been shown to inhibit tooth movement in OTM by downregulate STAT3 activity in periodontal ligament fibroblasts.^79,116^ In long bones, these inhibitors may lead to an increase in bone mass when target bone marrow macrophages. However, their effects on bone marrow stromal cells can contribute to a reduction in bone mass.^175^ These contrasting findings suggest that the potential adverse effects of AG490 and imatinib on alveolar bone, possibly inducing osteoporotic changes, require careful consideration when using STAT3 inhibitors. Targeted delivery systems may be necessary to achieve precise therapeutic outcomes. TA-21, which inhibits STAT3 specifically in regulatory T cells (Tregs), has been demonstrated to possess therapeutic potential for the treatment of rheumatoid arthritis, highlighting the nuanced role of STAT3 in immune modulation and its implications for bone.^176^ On the other hand, the STAT3 agonist, resveratrol, an anti-inflammatory agent, upregulates p-STAT3 while concurrently downregulating p-STAT1. This modulation promotes the transition from M1 to M2 macrophage polarization, which is advantageous for periodontal health and can help mitigate bone loss associated with inflammatory conditions.^173^ Additionally, fucoidan has been shown to enhance the phosphorylation levels of STAT3 in both unpolarized and reparative macrophages, thereby inhibiting OTM and promoting the stability of teeth following movement.^177^

Current research into therapies targeting JAK3 and STAT6 remains limited. However, two notable JAK3 inhibitors have demonstrated therapeutic effects in the treatment of arthritis, providing a foundational reference for potential therapies in craniofacial conditions. Eugenol has been shown to enhance the synthesis and secretion of type II collagen and proteoglycans in joint cartilage in murine models of arthritis. This effect is mediated through the inhibition of IL-1β-induced phosphorylation of JAK1 and STAT4, indicating its potential utility in modulating inflammatory responses in joints and possibly craniofacial contexts.^178^ Another JAK3 inhibitor, CS12192, functions by suppressing activation of CD4^+^ T cells, inhibiting Th17 cell function, and reducing RANKL-induced osteoclast formation, which collectively leads to diminished serum levels of pro-inflammatory cytokines.^179^ Han et al. reported the effects of a hexapeptide, WKYMVm, which activates the JAK1/STAT6 signaling. This activation promotes M2 macrophage polarization and facilitates angiogenesis and bone regeneration in models of femoral condyle injury, suggesting its potential applications in enhancing tissue repair and regeneration.^132^ Despite these advancements, there remains a significant gap in targeted drug studies involving other members of the JAK and STAT families, such as TYK2, STAT2, STAT4, and STAT5, particularly in craniofacial applications. While there has been substantial attention paid to the effects of these pathways in long bone-related diseases, their impact on craniofacial bones warrants further investigation.

### Proteins

Research on protein-targeted interventions within the JAK-STAT signaling as a therapeutic approach for craniofacial deformities remains sparse, with a predominant focus on the roles of STAT1 and STAT3. Recombinant ameloblastin has demonstrated efficacy in promoting bone healing in mandibular injury, facilitating the differentiation of human MSCs, osteoblasts, and osteoclasts, presumably by enhancing the expression of STAT1 and STAT2.^180^ Cytokines are frequently employed as targeted intervention agents. Jiang et al. revealed that in calvarial bone resorption associated with bone fragment granules, IL-10 downregulated JAK/STAT1 signaling while concurrently upregulating JAK/STAT3 in macrophages, thereby promoting the phenotypic transition from pro-inflammatory M1 macrophages to anti-inflammatory M2 macrophages.^181^ Furthermore, IL-27 has been shown to inhibit osteoblastic differentiation from human colony-forming unit granulocyte-macrophages (hCFU-GMs) and to suppress osteoclastic activity on dentin substrates by promoting STAT1 formation and phosphorylation while concurrently downregulating the transcription factor c-Fos, indicating an anti-inflammatory mechanism.^182^ Interestingly, application of the STAT1 inhibitor fludarabine and small interfering RNA targeting STAT1 partially reversed the effects elicited by IL-27.^182^ Small peptide molecules such as intermittent parathyroid hormone (iPTH) have been identified as activators of the STAT3/β-catenin pathway, leading to a reduction in alveolar bone loss during OTM in periodontal rat models, as reported by Zhang et al.^183^

### RNA

Research on the JAK-STAT signaling in the context of treating skeletal system abnormalities predominantly concentrates on the roles of JAK1, STAT1, and STAT3, particularly in relation to inflammatory processes. In models of periodontal disease, the *Mir338* cluster and miR-5134-5p, derived from osteoclast exosomes, have been implicated in the mechanisms underlying alveolar bone loss. The absence of the *Mir338* cluster has been shown to inhibit osteoclastogenesis via STAT1 suppression, resulting in heightened inflammatory responses and a shift in macrophage populations towards the M1 phenotype.^93^ Pan et al. found that miR-5134-5p exerted its effects by binding to JAK2, inhibiting osteoblast proliferation and differentiation through the JAK2/STAT3 signaling.^184^ Notably, the local application of antisense oligonucleotides targeting miR-338-3p or inhibitors of miR-5134-5p has proven effective in preventing alveolar bone loss.^93,184^ In the context of long bone arthritis, miR-17-5p has been identified as a regulator that targets both STAT3 and JAK1, leading to a significant reduction in the infiltration of B cells, T cells, macrophages, and multinucleated neutrophils within the synovium. This modulation alleviates structural damage and provides insights relevant to craniofacial inflammatory conditions.^185^ Regarding the osteogenic process, STAT1 has been found to negatively regulate osteogenic differentiation in stem cells derived from SHED cells. MiR-450a-5p and miR-28-5p interact with STAT1 mRNA to influence osteogenesis.^17^ In human MSCs, miR-224 targets Rac1 mRNA, thereby inhibiting Rac1 translation. The deficiency of Rac1 subsequently suppresses both the JAK/STAT3 and Wnt/β-catenin signaling pathways, promoting osteogenic differentiation.^186^ Additionally, Liu et al. have demonstrated that lncRNA Small nucleolar RNA host gene (SNHG)1 regulates the phosphorylation of STAT3 in human jaw bone marrow mesenchymal stem cells (h-JBMMSCs). This regulation leads to a reduction in reactive oxygen species (ROS) levels and modulates mitochondrial energy metabolism, ultimately enhancing cartilage regeneration.^187^

## Conclusion and perspectives

Dento-maxillofacial bones, which develop from both mesoderm and neural crest cells through endochondral and intramembranous ossification, present unique developmental characteristics that warrant focused investigation of JAK-STAT signaling functions in this region. Notably, mutations in JAK1, JAK2, STAT1, STAT3, STAT5, and STAT6 have been linked to skeletal abnormalities, with patients carrying STAT3 GOF, LOF, and STAT6 GOF mutations displaying specific dento-maxillofacial anomalies. Supplementary Table 1 provides a summary of gene-edited mouse models associated with the JAK-STAT signaling, reinforcing their relevance in disease modeling. The majority of existing research has mainly examined molecular changes at a systemic level, which limits the ability to elucidate the specific roles of particular cell types. Moreover, reliance on classical markers from long bone studies, such as *Osx, Col1*, and *Prx1*, has impeded the specificity needed for targeted investigations within craniofacial contexts. Nevertheless, the recent identification of distinct stem cell markers in the maxillofacial region presents a promising avenue for future exploration, particularly regarding the influence of the JAK-STAT signaling on these cells and its potential implications for stem cell therapies addressing craniofacial abnormalities.

The development of dento-maxillofacial complex originates from complex sources, and the function of JAK-STAT in long bones may not entirely align with its role in the complex. STAT1 is a prominent regulator, exhibiting different mechanisms in regulating endochondral ossification between cranial and long bones. JAK1, JAK2, STAT3, STAT5, and STAT6 demonstrate dual functions in bone formation and resorption during the development of both long bones and dento-maxillofacial bones. Notably, STAT1 and STAT3 also promote the function of tooth-forming cell populations which is a unique cellular cohort specific to the dental-periodontal complex. This specialization necessitates careful consideration when administering systemic inhibitors targeting STAT1 or STAT3 activity, as such treatments may potentially compromise dental development.

Furthermore, various therapeutic approaches targeting the JAK-STAT signaling have rapidly advanced, and precision medicine specifically aimed at this signaling holds great promise. Currently, small molecules such as Ruxolitinib and Fedratinib have been approved for the treatment of conditions like osteoporosis and osteoarthritis. However, due to differences in developmental origins, the conclusions drawn from studies on the JAK-STAT signaling in long bones and the efficacy of these drugs may not necessarily translate to craniofacial bone deformities. Thus, their mechanisms of action and safety profiles require further investigation.

In summary, the relative neglect of dento-maxillofacial abnormalities in research has resulted in a limited understanding of the role of the JAK-STAT signaling in this area. This gap highlights an important direction for both clinical and basic research within oral medicine.

## Supplementary information

Supplemental Material
