# Supplementary material for: JAK-STAT signaling: molecular mechanism and targeted treatment in dento-maxillofacial abnormalities
Source: Int J Oral Sci. 2026 Mar 5;18:24. doi: 10.1038/s41368-025-00399-z (PMC12960723; doi:10.1038/s41368-025-00399-z)
Supplement: Supplementary file 1 — Supplemental Material [file 41368_2025_399_MOESM1_ESM.docx]

Supplemental Material

Supplementary Table 1. Phenotypes of gene-edited mice with modified JAK-STAT signaling.

| Mouse Model | Effects on JAK-STAT Signaling | Phenotype | Reference |
| --- | --- | --- | --- |
| *Jak1*-/- | JAK1 deletion in all tissue | Perinatal mortality and reduced body size | ^1^ |
| *Jak1*H595D/+;I596I/+;Y597Y/+ | *Jak1* knock-in missense mutation in all tissue | Reduced body size, low weight, and skin hyperkeratosis. | ^2^ |
| *Ctsk^Cre^; Jak2^fl/fl^* | JAK2 deletion in osteoclasts | Reduced body size and decreased average femur length, but no significant changes in bone density or strength. | ^3^ |
| *Stat1^-/-^* | STAT1 deletion in all tissue | Increased cranial bone thickness in 14-week-old mice | ^4^ |
| *Osx^Cre^; Stat3^fl/fl^* | STAT3 deletion in osteoblast | Shortened roots of molars and incisors, with thinned dentin. | ^5,6^ |
| *Krt14^Cre^; Stat3^fl/fl^* | STAT3 deletion in epithelia including ameloblast progenitors and ameloblasts | Delayed enamel formation of the incisors. | ^7^ |
| *Prx1^Cre^; Stat3^fl/fl^* | STAT3 deletion in mesenchymal progenitors | Opened fontanelles of the skull, shortened limbs, bent forelimbs, and multiple spontaneous fractures in long bones. | ^8,9^ |
| *Dmp1^Cre^; Stat3^fl/fl^* | STAT3 deletion in osteocytes | Decreased osteoblastogenesis and increased osteoclastogenesis. No significant differences in trabecular or cortical bone. Diminished mechanoresponsiveness. | ^10^ |
| *Col1α1^Cre^; Stat3^fl/-^* | STAT3 deletion in osteoblast | Decreased osteoblast formation and increased osteoclast formation. No significant differences in femoral bone density, trabecular bone, or cortical bone. In vitro experiments indicated a reduction in bone formation induced by mechanical stress. | ^11^ |
| *Ctsk^Cre^; Stat3^fl/fl^* | STAT3 deletion in osteoclasts | Decreased bone mass in the femur and negatively regulated by estrogen. | ^12^ |
| *Stat5a^-/-^5b^-/-^* | STAT5A and STAT5B deletion in all tissue | Early mortality. | ^13^ |
| *Apn^Cre^; Stat5^fl/fl^* | STAT5 deletion in adipocytes | Enhanced adipogenic potential of BMSCs. | ^14^ |
| *Ctsk^Cre^; Stat5^fl/fl^* | STAT5 deletion in osteoclasts | Osteoporosis with increased resorptive capacity of osteoclasts. | ^15^ |
| *Mx1^Cre^;Stat5^fl/fl^* | STAT5 deletion in osteoclasts | Increased number of osteoclasts and decreased bone mass. | ^16^ |
| *Dmp1^Cre^;Socs3^fl/fl^* | SOCS3 deletion in osteocytes | Delayed reduction in cortical porosity and a transition from low to high density bone | ^17^ |

Supplementary Table 2. Molecules Targeting the JAK-STAT Signaling and their functions.

| Name | Classification | Selectivity | Potential Indication | Description | References |
| --- | --- | --- | --- | --- | --- |
| Baricitinib | Chemical and Natural Compound | JAK1、JAK2 | Rheumatoid Arthritis，Osteoporosis | Competitively bind to the ATP-binding site of both JAK1 and JAK2, suppress their phosphorylation. Improve osteoporosis and enhance bone density. | ^18^ |
| Tofacitinib | Chemical and Natural Compound | JAK1、JAK3 | Rheumatoid Arthritis | Competitively bind to the ATP-binding site of both JAK1 and JAK3, suppress their phosphorylation. Inhibit dysfunctional osteoclasts to reduce bone loss | ^19,20^ |
| GLPG0634 | Chemical and Natural Compound | JAK1、JAK2 | Arthritis | Selective inhibition of JAK1 over JAK2. Reduce differentiation of Th1, Th2, and Th17 cells, decrease cartilage and bone degradation | ^21^ |
| SHR0302 | Chemical and Natural Compound | JAK1 | Arthritis | Inhibit JAK1-STAT3 phosphorylation, suppress Th17 functionality and reduce B cells | ^22^ |
| Rhizoma Dioscoreae extract | Chemical and Natural Compound | JAK1？ | Ovariectomy-induced alveolar bone loss | Regulate JAK1/STAT3 signaling to protect against alveolar bone loss in ovariectomized rats | ^23^ |
| WKYMVm | Chemical and Natural Compound | JAK1、STAT6 | Bone regeneration | Activate JAK1/STAT6 pathway, promote angiogenesis and bone regeneration in mice with femoral condyle injuries. | ^24^ |
| Metformin | Chemical and Natural Compound | JAK2、STAT1 | Bone regeneration | Reduce phosphorylation levels of JAK2 and STAT1 to promote osteogenic differentiation of stem cells | ^25^ |
| Eupatilin | Chemical and Natural Compound | JAK2 | Ovariectomy-induced osteoporosis | Upregulate MiR-211-5p, which targets and inhibits JAK2, subsequently suppress STAT3 activation | ^26^ |
| A mixture of Humulus japonicus | Chemical and Natural Compound | JAK2、STAT5 | Bone development | Promote JAK2-STAT5 phosphorylation, promote IGF-1 synthesis and enhance growth of nasal tail length and femur-tibia length | ^27^ |
| THSG | Chemical and Natural Compound | JAK2、STAT3 | Tooth development | Promote JAK2-STAT3 phosphorylation, enhance the cell survival rate, telomerase activity, and embryoid body formation capacity | ^28^ |
| Eugenol | Chemical and Natural Compound | JAK3 | Arthritis | Inhibit JAK3/STAT4 phosphorylation, promote the synthesis and secretion of type II collagen and proteoglycans. | ^29^ |
| CS12192 | Chemical and Natural Compound | JAK3 | Bone regeneration | Inhibit osteoclast formation, reduce pro-inflammatory factor levels in serum. | ^30^ |
| Fludarabine | Chemical and Natural Compound | STAT1 | Bone fracture | Inhibit the formation of empty lacunae within the trabecular bone | ^31^ |
| Resveratrol | Chemical and Natural Compound | STAT1、STAT3 | Periodontitis | Activate STAT3 phosphorylation, inhibit STAT1 phosphorylation, and promote the M2 differentiation of macrophages. | ^32^ |
| Hydrogen sulfide | Chemical and Natural Compound | STAT1 | Orthodontic tooth movement | Promote STAT1 phosphorylation, induce M1 polarization of macrophages and osteoclast formation, and accelerate tooth movement | ^33^ |
| AG490 | Chemical and Natural Compound | STAT3 | Orthodontic tooth movement | Inhibit STAT3 in periodontal ligament fibroblasts and enhance the stability of teeth post-movement. | ^34,35^ |
| Imatinib | Chemical and Natural Compound | STAT3 | Orthodontic tooth movement | Inhibit STAT3 in periodontal ligament fibroblasts and enhance the stability of teeth post-movement. | ^34^ |
| STA-21 | Chemical and Natural Compound | STAT3 | Rheumatoid Arthritis | Inhibit STAT3 , promote the generation and function of Treg  cells and suppress the generation of Th17 cells and osteoclasts | ^36^ |
| Fucoidan | Chemical and Natural Compound | STAT3 | Orthodontic tooth movement | Promote STAT3 phosphorylation in unpolarized and repair-type macrophages,enhance the stability of teeth post-movement. | ^37^ |
| Recombinant ameloblastin | Proteins | STAT1、STAT2 | Bone regeneration | Enhanced STAT1 and STAT2, promote the differentiation of MSC, osteoblasts, and osteoclasts and facilitate the healing of mandibular injuries | ^38^ |
| Interleukin 10 | Proteins | STAT1、STAT3 | Bone regeneration | Inhibit STAT1 and promote STAT3 in macrophages, rescue calvarial bone resorption. | ^39^ |
| Interleukin 27 | Proteins | STAT1 | Bone regeneration | Promote STAT1, inhibit osteoblasts as well as the resorption of osteoclasts in dentin. | ^40^ |
| Parathyroid hormone | Proteins | STAT3 | Orthodontic tooth movement | Activate STAT3/β-catenin pathway, reduce alveolar bone loss during OTM with periodontitis. | ^41^ |
| miR-17-5p | RNA | JAK1、STAT3 | Arthritis | Significantly reduce the infiltration of B cells, T cells, macrophages, and multinucleated neutrophils in the synovium | ^42^ |
| miR-450a-5p | RNA | STAT1 | Tooth development | Target STAT1 mRNA, promote osteogenesis. | ^43^ |
| miR-28-5p | RNA | STAT1 | Tooth development | Target STAT1 mRNA, promote osteogenesis. | ^43^ |
| miR-224 | RNA | STAT3 | Bone development | Inhibit the translation of Rac1 , suppress  JAK/STAT3 signaling and promote osteogenic differentiation. | ^44^ |
| lncRNA SNHG1 | RNA | STAT3 | Bone regeneration | Regulate STAT3 phosphorylation, reduce  ROS levels and promote cartilage regeneration | ^45^ |

References

1. Rodig, S. J. *et al.* Disruption of the Jak1 gene demonstrates obligatory and nonredundant roles of the Jaks in cytokine-induced biologic responses. *Cell* 93, 373–383 (1998).

2. Takeichi, T. *et al.* Autoinflammatory Keratinization Disease With Hepatitis and Autism Reveals Roles for JAK1 Kinase Hyperactivity in Autoinflammation. *Front Immunol* 12, 737747 (2021).

3. Dodington, D. W. *et al.* JAK2-IGF1 axis in osteoclasts regulates postnatal growth in mice. *JCI Insight* 6, e137045, 137045 (2021).

4. Meraz, M. A. *et al.* Targeted disruption of the Stat1 gene in mice reveals unexpected physiologic specificity in the JAK-STAT signaling pathway. *Cell* 84, 431–442 (1996).

5. Chan, L. *et al.* Loss of Stat3 in Osterix+ cells impairs dental hard tissues development. *Cell Biosci* 13, 75 (2023).

6. Zhou, S. *et al.* STAT3 is critical for skeletal development and bone homeostasis by regulating osteogenesis. *Nat Commun* 12, 6891 (2021).

7. Zhang, B. *et al.* The Role of Epithelial Stat3 in Amelogenesis during Mouse Incisor Renewal. *Cells Tissues Organs* 205, 63–71 (2018).

8. Yadav, P. S. *et al.* Stat3 loss in mesenchymal progenitors causes Job syndrome-like skeletal defects by reducing Wnt/β-catenin signaling. *Proc Natl Acad Sci U S A* 118, e2020100118 (2021).

9. Huang, Z. *et al.* Loss of signal transducer and activator of transcription 3 impaired the osteogenesis of mesenchymal progenitor cells in vivo and in vitro. *Cell Biosci* 11, 172 (2021).

10. Corry, K. A. *et al.* Stat3 in osteocytes mediates osteogenic response to loading. *Bone Rep* 11, 100218 (2019).

11. Itoh, S. *et al.* A critical role for interleukin-6 family-mediated Stat3 activation in osteoblast differentiation and bone formation. *Bone* 39, 505–512 (2006).

12. Davidson, R. K. *et al.* The loss of STAT3 in mature osteoclasts has detrimental effects on bone structure. *PLoS One* 15, e0236891 (2020).

13. Socolovsky, M., Fallon, A. E., Wang, S., Brugnara, C. & Lodish, H. F. Fetal anemia and apoptosis of red cell progenitors in Stat5a-/-5b-/- mice: a direct role for Stat5 in Bcl-X(L) induction. *Cell* 98, 181–191 (1999).

14. Seong, S. *et al.* Alternative regulatory mechanism for the maintenance of bone homeostasis via STAT5-mediated regulation of the differentiation of BMSCs into adipocytes. *Exp Mol Med* 53, 848–863 (2021).

15. Hirose, J. *et al.* Bone resorption is regulated by cell-autonomous negative feedback loop of Stat5-Dusp axis in the osteoclast. *J Exp Med* 211, 153–163 (2014).

16. J, L. *et al.* STAT5 is a key transcription factor for IL-3-mediated inhibition of RANKL-induced osteoclastogenesis. *Scientific reports* 6, (2016).

17. Walker, E. C. *et al.* Cortical bone maturation in mice requires SOCS3 suppression of gp130/STAT3 signalling in osteocytes. *Elife* 9, e56666 (2020).

18. Sanchez, G. A. M. *et al.* JAK1/2 inhibition with baricitinib in the treatment of autoinflammatory interferonopathies. *J Clin Invest* 128, 3041–3052 (2018).

19. Orsolini, G., Bertoldi, I. & Rossini, M. Osteoimmunology in rheumatoid and psoriatic arthritis: potential effects of tofacitinib on bone involvement. *Clin Rheumatol* 39, 727–736 (2020).

20. Chiu, Y.-S. *et al.* The JAK inhibitor Tofacitinib inhibits structural damage in osteoarthritis by modulating JAK1/TNF-alpha/IL-6 signaling through Mir-149-5p. *Bone* 151, 116024 (2021).

21. Van Rompaey, L. *et al.* Preclinical characterization of GLPG0634, a selective inhibitor of JAK1, for the treatment of inflammatory diseases. *J Immunol* 191, 3568–3577 (2013).

22. Wu, H. *et al.* JAK1-STAT3 blockade by JAK inhibitor SHR0302 attenuates inflammatory responses of adjuvant-induced arthritis rats and decreases Th17 and total B cells. *Joint Bone Spine* 83, 525–532 (2016).

23. Zhang, Z. *et al.* Rhizoma Dioscoreae extract protects against alveolar bone loss in ovariectomized rats via microRNAs regulation. *Nutrients* 7, 1333–1351 (2015).

24. Han, X. *et al.* Hexapeptide induces M2 macrophage polarization via the JAK1/STAT6 pathway to promote angiogenesis in bone repair. *Exp Cell Res* 413, 113064 (2022).

25. Lin, R. *et al.* Metformin attenuates diabetes-induced osteopenia in rats is associated with down-regulation of the RAGE-JAK2-STAT1 signal axis. *J Orthop Translat* 40, 37–48 (2023).

26. Hong, L. & Yang, C. Eupatilin ameliorates postmenopausal osteoporosis via elevating microRNA-211-5p and repressing JAK2/STAT3 pathway. *Environ Toxicol* 39, 2218–2228 (2024).

27. Kim, O.-K. *et al.* A Mixture of Humulus japonicus Increases Longitudinal Bone Growth Rate in Sprague Dawley Rats. *Nutrients* 12, 2625 (2020).

28. Huang, Y.-W. *et al.* 2,3,5,4’-tetrahydroxystilbene-2-O-b-D-glucoside triggers the pluripotent-like possibility of dental pulp stem cells by activating the JAK2/STAT3 axis: Preliminary observations. *J Dent Sci* 16, 599–607 (2021).

29. Wu, Z., Wang, Y., Yan, G. & Wu, C. Eugenol protects chondrocytes and articular cartilage by downregulating the JAK3/STAT4 signaling pathway. *J Orthop Res* 41, 747–758 (2023).

30. Shan, S. *et al.* Therapeutic treatment of a novel selective JAK3/JAK1/TBK1 inhibitor, CS12192, in rat and mouse models of rheumatoid arthritis. *Int Immunopharmacol* 77, 105914 (2019).

31. Lin, J. *et al.* Downregulating STAT1/caspase-3 signaling with fludarabine to alleviate progression in a rat model of steroid-induced avascular necrosis of the femoral head. *J Biochem Mol Toxicol* 33, e22265 (2019).

32. Shi, J. *et al.* Remodeling immune microenvironment in periodontitis using resveratrol liposomes as an antibiotic-free therapeutic strategy. *J Nanobiotechnology* 19, 429 (2021).

33. He, D. *et al.* Mechanical load-induced H2S production by periodontal ligament stem cells activates M1 macrophages to promote bone remodeling and tooth movement via STAT1. *Stem Cell Res Ther* 11, 112 (2020).

34. Jin, Y. *et al.* Tensile force-induced PDGF-BB/PDGFRβ signals in periodontal ligament fibroblasts activate JAK2/STAT3 for orthodontic tooth movement. *Sci Rep* 10, 11269 (2020).

35. Gong, X. *et al.* Osteoblastic STAT3 Is Crucial for Orthodontic Force Driving Alveolar Bone Remodeling and Tooth Movement. *Journal of Bone and Mineral Research* 38, 214–227 (2023).

36. Park, J.-S. *et al.* STA-21, a promising STAT-3 inhibitor that reciprocally regulates Th17 and Treg cells, inhibits osteoclastogenesis in mice and humans and alleviates autoimmune inflammation in an experimental model of rheumatoid arthritis. *Arthritis Rheumatol* 66, 918–929 (2014).

37. Zhang, S. *et al.* Fucoidan inhibits tooth movement by promoting restorative macrophage polarization through the STAT3 pathway. *J Cell Physiol* 235, 5938–5950 (2020).

38. Tamburstuen, M. V. *et al.* Ameloblastin promotes bone growth by enhancing proliferation of progenitor cells and by stimulating immunoregulators. *Eur J Oral Sci* 118, 451–459 (2010).

39. Jiang, J. *et al.* Macrophage Polarization in IL-10 Treatment of Particle-Induced Inflammation and Osteolysis. *Am J Pathol* 186, 57–66 (2016).

40. Furukawa, M. *et al.* IL-27 abrogates receptor activator of NF-kappa B ligand-mediated osteoclastogenesis of human granulocyte-macrophage colony-forming unit cells through STAT1-dependent inhibition of c-Fos. *J Immunol* 183, 2397–2406 (2009).

41. Zhang, C. *et al.* Parathyroid hormone increases alveolar bone homoeostasis during orthodontic tooth movement in rats with periodontitis via crosstalk between STAT3 and β-catenin. *Int J Oral Sci* 12, 38 (2020).

42. Najm, A. *et al.* MicroRNA-17-5p Reduces Inflammation and Bone Erosions in Mice With Collagen-Induced Arthritis and Directly Targets the JAK/STAT Pathway in Rheumatoid Arthritis Fibroblast-like Synoviocytes. *Arthritis Rheumatol* 72, 2030–2039 (2020).

43. Dernowsek, J. A. *et al.* Posttranscriptional Interaction Between miR-450a-5p and miR-28-5p and STAT1 mRNA Triggers Osteoblastic Differentiation of Human Mesenchymal Stem Cells. *J Cell Biochem* 118, 4045–4062 (2017).

44. Cai, Q. *et al.* MicroRNA-224 enhances the osteoblastic differentiation of hMSCs via Rac1. *Cell Biochem Funct* 37, 62–71 (2019).

45. Liu, H., Liu, H., Yang, Q. & Fan, Z. LncRNA SNHG1 enhances cartilage regeneration by modulating chondrogenic differentiation and angiogenesis potentials of JBMMSCs via mitochondrial function regulation. *Stem Cell Res Ther* 15, 177 (2024).
